# ONLY IN DREAMS: a case report of sleep deprivation psychosis

**DOI:** 10.1192/j.eurpsy.2022.1996

**Published:** 2022-09-01

**Authors:** J. Brás, A. Costa, R. Sousa, R. Vaz, J. Martins, E. Almeida, J. Abreu, A. Costa

**Affiliations:** Centro Hospitalar Tondela-Viseu , Departamento De Psiquiatria E Saúde Mental, Viseu, Portugal

**Keywords:** clinical case, Sleep deprivation, Psychosis, Insomnia

## Abstract

**Introduction:**

Sleep is essential for an adequate neurobiological functioning, being implicated in several cognitive functions. Even in healthy individuals, sleep deprivation can lead to a number of psychopathological changes, including perceptual distortions, hallucinations and delusions. Thus, the resulting clinical picture may be similar to a psychotic disorder.

**Objectives:**

To present a clinical case of psychotic symptomatology induced by sleep deprivation.

**Methods:**

Patient’s clinical file consultation and literature review using the search engine Pubmed® and the keywords: “sleep deprivation”, “sleep loss” and “psychosis”.

**Results:**

We present the case of a 41-year-old woman with a history of an episode of mood changes with psychotic symptoms that was preceded by a period of total insomnia. No psychotropic drugs since then and no relapses. In May 2020, she was admitted in psychiatry department due to clinical picture composed by significant psychomotor slowing, drowsiness, slowed speech, verbal visual, tactile and auditory hallucinations accompanied by grandiose delusions. These symptoms were preceded by total insomnia with one week of duration. In the hospital was administered quetiapine 100mg and lorazepam 2.5mg to aid in the recovery of sleep deprivation and concomitantly aripiprazole 15mg was prescribed. The patient presented a rapid and significant clinical improvement. Currently, it is without any type of medication and without psychopathological changes.

**Conclusions:**

The clinical picture present in this case report was triggered after a significant period of sleep deprivation. Thus, it illustrates the role that sleep has in the development of psychiatric symptomatology, sometimes difficult to differentiate from psychiatric disorders.

**Disclosure:**

No significant relationships.

